# Supplementary material for: FBN-1, a fibrillin-related protein, is required for resistance of the epidermis to mechanical deformation during C. elegans embryogenesis
Source: eLife. 2015 Mar 23;4:e06565. doi: 10.7554/eLife.06565 (PMC4395870; doi:10.7554/eLife.06565)
Supplement: Supplementary file 1. — Percentage of Pin Animals. DOI: http://dx.doi.org/10.7554/eLife.06565.014 [file elife06565s001.pdf]

Supplementary File 1. Percentage of Pin Animals

Genotype	# Pin	N	% Pin	95% CI
N2	0	>500	0	ND
sym-3(mn618)	3	114	2.6	0.6–7.8
sym-4 (mn619)	76	539	14.1	11.4–17.3
mec-8(u74)	5	126	4.0	1.5–9.2
mec-8; sym-3[a]	131	132	99	95.4–100
mec-8; sym-4	267	280	95.3	92.1–97.3
N2 control*(RNAi)*	0	42	0	ND
N2 *fbn-1(RNAi)*	0	113	0	ND
sym-3; control*(RNAi)*	2	123	1.6	0.1–6.1
sym-3; fbn-1(RNAi)	80	135	59.2	50.8–67.2 (±8.2)
sym-4; control*(RNAi)*	4	86	4.7	1.5–11.4(±5.1)
sym-4; fbn-1(RNAi)	44	135	32.6	25.2–40.9(±7.9)
mec-8; control*(RNAi)*	10	132	7.6	4.2–13.4(±4.6)
mec-8; fbn-1(RNAi)	50	196	25.5	19.9–32.0(±6.1)
lin-35; control*(RNAi)*	0	86	0	ND
lin-35; fbn-1(RNAi)	23	68	33.8	23.7–45.7(±11.0)
lin-35; sym-3; control*(RNAi)*	6	73	8.2	3.5–17.1(±6.8)
lin-35; sym-3; fbn-1(RNAi)	66	75	88	78.5–93.8(±7.7)
lin-35; sym-4; control*(RNAi)*	14	116	12.1	7.2–19.4(±6.1)
lin-35; sym-4; fbn-1(RNAi)	113	120	94.2	88.2–97.4(±4.6)
mec-8; rrf-3; control*(RNAi)*	5	94	5.3	2.0–12.2(±5.1)
mec-8; rrf-3; fbn-1(RNAi)	35	42	83.3	69.1–92.0(±11.5)
N2	0	>500	0	ND

sym-3	3	114	2.6	0.6–7.8
sym-4	76	539	14.1	11.4–17.3
fbn-1(ns67)	54	124	43.5	35.1–52.3
fbn-1(ns283)	66	154	42.9	35.3–50.8
fbn-1(tm290)	949	187	19.7	17.3–22.4
fbn-1(ns67); sym-3	89	90	98.9	93.4–100
fbn-1(ns67); sym-4	86	86	100	95–100
mec-8; fbn-1(n67)	58	60	96.7	88–99.7
fbn-1(tm290); sym-3	96	96	100	95.4–100
fbn-1(tm290); sym-4	71	71	100	93.9–100
mec-8; fbn-1(tm290)	155	155	100	97.1–100
N2	0	>500	0	ND
mec-8; sym-4	267	280	95.3	92.1–97.3
mec-8; sym-4; fdEx251	42	104	40.1	31.5–50.0
mec-8; sym-4; fdEx254	63	103	61.1	51.5–70.0
N2; control*(RNAi)*	0	42	0	0–10
N2; *fbn-1(RNAi)*	0	113	0	0–3.9
mec-8; control*(RNAi)*	10	132	7.6	4.2–13.4(±4.6)
mec-8; fbn-1(RNAi)	50	196	25.5	19.9–32.0(±6.1)
rde-1; control*(RNAi-Hyp)* [b]	0	58	0	ND
rde-1; fbn-1(RNAi-Hyp) [b]	0	93	0	ND
mec-8; rde-1; control*(RNAi-Hyp)* [b]	14	177	7.9	4.7–12.9
mec-8; rde-1; fbn-1(RNAi-Hyp) [b]	31	159	19.5	14–26.4

[a]Because this strain (WY893) gives rise to a high frequency of viable *mnEx169*(–) progeny in the first generation following loss of the array (F1 escapers), next-generation progeny (F2) from *mnEx169*(–) F1

parents were scored. [b]Strains were derived from NR222. CI, confidence interval; ND, not determined. CIs were calculated using the Agresti-Coull method.
