# Supplementary material for: FBN-1, a fibrillin-related protein, is required for resistance of the epidermis to mechanical deformation during C. elegans embryogenesis
Source: eLife. 2015 Mar 23;4:e06565. doi: 10.7554/eLife.06565 (PMC4395870; doi:10.7554/eLife.06565)
Supplement: Supplementary file 2. — Statistical analysis of FRET data. DOI: http://dx.doi.org/10.7554/eLife.06565.015 [file elife06565s002.pdf]

Supplementary File 2 (A–D). Statistical analysis of FRET data.

A. T-test values for UNC-70(TSMod) strains.

	TS(Mod) N2 Comma SDR	TS(Mod) N2 Comma Out-SDR	TS(Mod) N2 1.5-fold SDR	TS(Mod) N2 1.5-fold Out-SDR	TS(Mod) *pha-1* Comma SDR	TS(Mod) *pha-1* Comma Out-SDR	TS(Mod) *pha-1* 1.5-fold SDR	TS(Mod) *pha-1* 1.5-fold Out-SDR
**TS(Mod) N2 Comma SDR**		**P=0.5572** P=0.1683	**P=0.0001**	**P=0.7456**	**P=1.0000**	**P=0.4204**	**P=0.7089**	**P=0.2554**
**TS(Mod) N2 Comma Out-SDR**			**P=0.0004**	**P=0.7626**	**P=0.5879**	**P=0.2288**	**P=0.5578**	**P=0.1235**
**TS(Mod) N2 1.5-fold SDR**				**P=0.0008** P<0.0001	**P=0.0002**	**P<0.0001**	**P<0.0001**	**P<0.0001**
**TS(Mod) N2 1.5-fold Out-SDR**					**P=0.7650**	**P=0.2868**	**P=0.7589**	**P=0.1593**
**TS(Mod) *pha-1* Comma SDR**						**P=0.3737** P=0.1637	**P=0.9745**	**P=0.3132**
**TS(Mod) *pha-1* Comma Out-SDR**							P=0.3853	P=0.8861
**TS(Mod) *pha-1* 1.5-fold SDR**								**P=0.2421** P=0.0291
**TS(Mod) *pha-1* 1.5-fold Out-SDR**								

P-values in bold are derived from a 2-tailed non-paired T-test. Non-bold P-values are derived from a 2-tailed paired T-test (where applicable). Yellow background indicates very high statistical significance (P<0.001). Green background indicates marginal statistical significance (0.01<P<0.05). Tests were carried out in the N2 (wild-type) strain or in *pha-1(tm3671)* mutants, as indicated. All tests were performed with the UNC-70(TSMod).

B. T-test values for controls.

	No Force cont. N2 Comma SDR	No Force cont. N2 Comma Out-SDR	No Force cont. N2 1.5-fold SDR	No Force cont. N2 1.5-fold Out-SDR	5aa-cont. N2 1.5-fold SDR	5aa-cont. N2 1.5-fold Out-SDR	TRAF-cont. N2 1.5-fold SDR	TRAF-cont. N2 1.5-fold Out-SDR
**No Force cont. N2 Comma SDR**		**P=0.6648**<br>P=0.1508	**P=0.1841**	**P=0.3688**				
**No Force cont. N2 Comma Out-SDR**			**P=0.0726**	**P=0.1058**				
**No Force cont. N2 1.5-fold SDR**				**P=0.6630**<br>P=0.0140				
**No Force cont. N2 1.5-fold Out-SDR**								
**5aa-cont. N2 1.5-fold SDR**						**P=0.6618**<br>P=0.0875		
**5aa-cont. N2 1.5-fold Out-SDR**								
**TRAF-cont. N2 1.5-fold SDR**								**P=0.2452**<br>P=0.1080
**TRAF-cont. N2 1.5-fold Out-SDR**								

P-values in bold are derived from a 2-tailed non-paired T-test. Non-bold P-values are derived from a 2-tailed paired T-test (where applicable). All tests were carried out in the N2 (wild-type) strain. Green background indicates marginal statistical significance (0.01<P<0.05). Tests were performed with the UNC-70(N-TSMod) (No Force Control), the 5aa spacer, or the TRAF spacer.

C. T-test values for UNC-70(TSMod) versus UNC-70(N-TSMod).

	No Force cont. N2 Comma SDR	No Force cont. N2 Comma Out-SDR	No Force cont. N2 1.5-fold SDR	No Force cont. N2 1.5-fold Out-SDR	TS(Mod) N2 Comma SDR	TS(Mod) N2 Comma Out-SDR	TS(Mod) N2 1.5-fold SDR	TS(Mod) N2 1.5-fold Out-SDR
**No Force cont. N2 Comma SDR**					**P=0.0149**	**P=0.0087**	**P<0.0001**	**P=0.0079**
**No Force cont. N2 Comma Out-SDR**					**P=0.0049**	**P=0.0032**	**P<0.0001**	**P=0.0025**
**No Force cont. N2 1.5-fold SDR**					**P=0.1168**	**P=0.0415**	**P<0.0001**	**P=0.0529**
**No Force cont. N2 1.5-fold Out-SDR**					**P=0.0736**	**P=0.0434**	**P<0.0001**	**P=0.0307**
**TS(Mod) N2 Comma SDR**								
**TS(Mod) N2 Comma Out-SDR**								
**TS(Mod) N2 1.5-fold SDR**								
**TS(Mod) N2 1.5-fold Out-SDR**								

P-values in bold are derived from a 2-tailed non-paired T-test.). All tests were carried out in the N2 (wild-type) strain. Green background indicates marginal statistical significance (0.01<P<0.05). Red background indicates high statistical significance (P<0.01). Yellow background indicates very high statistical significance (P<0.001). The detected differences between UNC-70(TSMod) and UNC-70(N-TSMod) (No Force control) in SDR and Out-SDR regions in early comma stages and in the Out-SDR region at the 1.5-fold stage may indicate that these regions are normally under low-to-moderate levels of tension that are independent of pharyngeal attachment, which are detected by the UNC-70(TSMod).

D. Data used for calculations.

Strain Background	TS module	Stage	Region	Mean	SD	N
N2	UNC-70(TSMod)	Early Comma	SDR	0.2591	0.0673	27
N2	UNC-70(TSMod)	Early Comma	Non-SDR	0.2467	0.0858	27
N2	UNC-70(TSMod)	1.5-Fold	SDR	0.1973	0.0517	35
N2	UNC-70(TSMod)	1.5-Fold	Non-SDR	0.2525	0.0772	35
pha-1(tm3671)	UNC-70(TSMod)	Early Comma	SDR	0.2591	0.0630	20
pha-1(tm3671)	UNC-70(TSMod)	Early Comma	Non-SDR	0.2762	0.0764	20
pha-1(tm3671)	UNC-70(TSMod)	1.5-Fold	SDR	0.2585	0.0664	31
pha-1(tm3671)	UNC-70(TSMod)	1.5-Fold	Non-SDR	0.2799	0.0759	31
N2	UNC-70(N-TSMod)	Early Comma	SDR	0.3048	0.0593	23
N2	UNC-70(N-TSMod)	Early Comma	Non-SDR	0.3125	0.0604	23
N2	UNC-70(N-TSMod)	1.5-Fold	SDR	0.2840	0.0576	37
N2	UNC-70(N-TSMod)	1.5-Fold	Non-SDR	0.2901	0.0622	37
N2	UNC-70(TRAF)	1.5-Fold	SDR	0.1068	0.0548	11
N2	UNC-70(TRAF)	1.5-Fold	Non-SDR	0.1337	0.0455	11
N2	UNC-70(5aa)	1.5-Fold	SDR	0.4241	0.0801	21
N2	UNC-70(5aa)	1.5-Fold	Non-SDR	0.4356	0.0888	21
